# Effects of different iron treatments on wine grape berry quality and peel flavonoid contents

**DOI:** 10.1002/fsn3.2957

**Published:** 2022-07-02

**Authors:** Shu Zhang, Heting Chen, Ming Gao, Chaofeng Gu, Rui Wang

**Affiliations:** ^1^ College of Agronomy Ningxia University Yinchuan P.R. China; ^2^ Ningxia Grape and Wine Research Institute Yinchuan P.R. China; ^3^ China Wine Industry Technology Institute Yinchuan P.R. China

**Keywords:** anthocyanin, flavonoid, iron, wine grape quality

## Abstract

In this study, eight‐year‐old wine grape plants (Cabernet Sauvignon) were subjected to five different iron treatments: ferrous sulfate, ferric ethylenediaminetetraacetic acid (EDTA‐Fe), ferric citrate, ferric gluconate, and ferric sugar alcohol, and conventional fertilization. Foliar spraying with clear water was used as the control treatment. The effects of different iron treatments on berry quality and flavonoid accumulation in grape peels were explored. All five iron treatments affected the sugar, acid, and peel flavonoid contents of grape berries, but the contents varied greatly among the different iron treatments. Foliar spraying with iron increased berry sugar content and reduced acid content. In addition, foliar spraying with ferrous sulfate, EDTA‐Fe, ferric gluconate, and ferric sugar alcohol reduced the total anthocyanin, flavanol, and flavonol contents in the peel. The unique flavonoid monomer content of the peel was significantly higher under ferric citrate treatment than under the control and other iron treatments. Moreover, the results showed that foliar spraying with ferric citrate balanced the berry sugar–acid ratio and also increased the anthocyanin, flavanol, and flavonol contents of the grape peel, thereby improving the overall nutritional status of the berries and the final wine quality. The results obtained in this study demonstrate that different iron treatments could improve grape berry quality and clarify the effects of different exogenous iron treatments.

## Graphical abstract

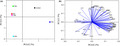

## 1 INTRODUCTION

The eastern foothills of Helan Mountain in Ningxia are among the best ecological regions for wine grapes in China. However, the fertilization method used for wine grape cultivation is inclined to supplement traditional macronutrient fertilizers, ignoring the importance of trace elements. The mineral nutrient availability in the alkaline soil in this area is low, the effective iron content is far lower than the national average, and the mobility of iron in plants is not strong, making it difficult for various elements that were originally insufficient to meet the nutritional requirements for the normal growth of grapes.

Foliar fertilization is one of the main methods used to improve the berry yield and quality during grape cultivation. Foliar application is the most rapid and effective method for satisfying the specific nutritional needs of plants (Fernandez et al., 2009). The effect of foliar iron application on the composition of grapes is highly dependent on the grape variety and the duration and form of iron used (Abadia et al., 2002; Alvarez‐Fernàndez et al., 2006; Yunta et al., 2013). Iron fertilizers can be divided into three categories: inorganic, organic compound, and chelated iron fertilizers. Inorganic iron fertilizer is inexpensive and has a fast fertilizer effect, but it is easily oxidized, resulting in an unsustainable fertilizer effect. Ferrous sulfate is the most common fertilizer. Organic compound iron fertilizers are relatively stable and easily degradable, and the most common ones are ferric gluconate and ferric sugar alcohol. The chelated iron fertilizers are mainly ferric ethylenediaminetetraacetic acid (EDTA‐Fe), ethylenediamine‐N,N′‐bis(2‐hydroxyphenylacetic acid) (EDDHA‐Fe), and ferric citrate. Furthermore, studies have shown that the application of chelated iron can significantly increase the soluble sugar and phenolic acid contents of grapes under alkaline soil conditions (Karimi et al., 2019).

The quality of grapes is related to the balance between primary and secondary metabolites. Glucose and fructose are the main sugars in berries, and the contents of these primary metabolites are affected by variety, climate, and nutritional status, while the accumulation of sugar in berries can improve the volatile aromatic compound content (Ali et al., 2010; Zheng et al., 2009). Phenols are important components that determine the quality of grape berries and have various functions in plants, including as antioxidants, protecting against ultraviolet (UV) radiation, and combating pathogenic infections (Tian et al., 2019). Studies have shown that iron is related to the function of phenolic synthase; thus, iron deficiency affects the synthesis of phenolic compounds via the shikimate pathway (Bavaresco et al., 2005). In addition, iron concentration can affect pectin degradation and antioxidant capacity of phenolic compounds (Vidot et al., 2020). Many phenolic compounds are found in grapes at high concentrations. These phenolic compounds can be divided into flavonoids and nonflavonoids, where flavonoids comprise flavanols, flavonols, and anthocyanins, and nonflavonoids include resveratrol, benzoic acid, and cinnamic acid (Liang et al., 2013). The expression levels of genes related to anthocyanin biosynthesis are affected by developmental and environmental factors, including temperature, light, sugar content, and mineral content. Karimi et al. (2019) found that exogenous iron could regulate the anthocyanin content of berries. Zheng et al. (2009) showed that carbohydrates can promote the expression of flavanone 3‐hydroxylase (EC 1.14.11.9), which is a key enzyme in anthocyanin synthesis, thereby increasing berry anthocyanin content. Flavonols are produced by the flavonoid biosynthesis pathway and mainly comprise quercetin, myricetin, kaempferol, and isorhamnetin (Mattivi et al., 2006). Flavan‐3‐alcohol is a basic component of proanthocyanidins and condensed tannins, including catechin, epicatechin, gallocatechin, epigallocatechin, and epigallocatechin gallate. Flavonoids and flavanols are mainly distributed in the grape seeds, peel, and berry stems. Flavanols have important effects on astringency, bitterness, and structure of wine (Zimman et al., 2002). Foliar spraying with iron can increase the anthocyanin (Shi et al., 2017), flavonol, and flavanol (Shi et al., 2018) contents of grape berries. However, previous studies have not comprehensively investigated the effects of different forms of iron on the flavonoid content of grapes.

In this study, we investigated the effects of foliar treatment with different forms of iron on berry quality and flavonoid accumulation in the peel of Cabernet Sauvignon grapes, thereby providing a theoretical basis for improving wine grape cultivation in alkaline soil at the eastern foothills of the Helan Mountain in China.

## 2 MATERIALS AND METHODS

### 2.1 Test materials and experimental design

The experiment was conducted from April to October, 2021 at Lilan Winery (105°58′20′′E, 38°16′38′′N), which is in a wine grape‐producing area at the eastern foothills of the Helan Mountain in China. The study site was located at an altitude of 1129 m, with an annual average precipitation of 190 mm and a frost‐free period of 180 days. Eight‐year‐old Cabernet Sauvignon grape vines were used in this study. The vine shape was “厂” and the row spacing was 0.6 × 3.5 m. The basal fertilizer, comprising organic sheep manure fertilizer, was ditched in early May and applied once at 10,500 kg hm^−2^ by drip irrigation. The soil type was calcareous gravel. Table 1 lists the chemical characteristics of the soil before the start of the experiment.

**TABLE 1:** Chemical characteristics of the soil before the trial commenced (g kg^−1^)

Soil depth (m)	pH	N	P	K	Fe
0–0.2	8.45	5.17	2.54	7.22	16.32
0.2–0.4	8.55	3.25	2.12	6.90	15.41
0.4–0.6	8.61	3.15	2.07	6.82	14.65

The experiment had a randomized block design with six treatments and three replicates for each treatment, with a total of 18 cells, and each cell area was 10.5 m^2^. Each iron fertilizer treatment was sprayed three times (on July 12, July 27, and August 11) with an electric sprayer at the grape expansion and color‐changing stages, where a 5 L solution of each treatment was applied. Table 2 shows the total amounts of iron received for each treatment after the three applications. Excluding the different forms of iron fertilizer, all other treatments and management measures were consistent with those used in the vineyard. During the optimal grape harvest period, 15 representative grapes were randomly selected from each treatment and transported rapidly to the laboratory. One hundred grapes were immediately frozen in liquid nitrogen and ground to determine berry quality. For each treatment, 30 grapes were randomly selected, washed with distilled water, peeled, frozen in liquid nitrogen, and stored at −80°C to determine the peel metabolites.

**TABLE 2:** Iron treatments applied to grapevines

Indexes	Form of iron fertilizer	Solution concentration (%)	Iron fertilizer application rate (kg hm^−2^)	Iron dosage (kg hm^−2^)
Control	–		–	–
FB1	Ferrous sulfate	0.09	4.44	0.89
FB2	EDTA‐Fe	0.15	7.14	0.89
FB3	Ferric citrate	0.08	3.99	0.89
FB4	Ferric gluconate	0.16	7.76	0.89
FB5	Ferric sugar alcohol	0.19	8.93	0.89

*Note*: Ferrous sulfate (Fe 20.10%), EDTA‐Fe (Fe 12.50%), ferric citrate (Fe 22.40%), ferric gluconate (Fe 11.50%), and ferric sugar alcohol (Fe 100 g L^−1^).

### 2.2 Determination of grape berry quality

Frozen grape berries were ground to determine the total soluble solids (TSS), reducing sugar, and titratable acid contents (TAC). The TSS content was determined using a handheld glucose meter. The reducing sugar content was determined using the 3,5‐dinitrosalicylic acid method. TAC was determined by titration using standard 0.1 mol L^−1^ NaOH (endpoint pH 8.2) (Jin et al., 2016; Ma et al., 2019; Wang et al., 2019).

### 2.3 Extraction of anthocyanin and flavonoid compounds from peel

Grape peel samples were vacuum dried and frozen for 24 h in a lyophilizer (Scientz‐100F) and then ground (30 Hz, 1.5 min) into powder using a grinder (MM 400, Retsch). The powdered peel (100 mg) was extracted with 70% methanol solution (1.2 ml) before mixing six times in a vortex shaker for 30 s each at 30 min intervals and standing overnight at 4°C. The homogenate was centrifuged at 12,000 rpm (revolutions per minute) for 10 min and the supernatant was aspirated and filtered through a microporous membrane (pore size = 0.22 μm) for UPLC–MS/MS (ultrahigh performance liquid chromatography tandem spectrometry) analysis. Three independent extractions were performed for each treatment group.

#### 2.3.1 UPLC–MS/MS conditions

The sample extracts were analyzed using an UPLC–ESI–MS/MS system (ultrahigh performance liquid chromatography–electrospray ionization tandem mass spectrometry) (UPLC, SHIMADZU Nexera X2, www.shimadzu.com.cn/; MS, Applied Biosystems 4500 Q TRAP, www.appliedbiosystems.com.cn/). The analytical conditions were as follows: UPLC column, Agilent SB‐C18 (1.8 μm, 2.1 × 100 mm); mobile phase solvent A comprising pure water with 0.1% formic acid and solvent B comprising acetonitrile with 0.1% formic acid. Sample measurements were performed with a gradient program using starting conditions of 95% A/5% B. After 9 min, a linear gradient of 5% A/95% B was programmed, and a composition of 5% A/95% B was maintained for 1 min. Subsequently, the composition was adjusted to 95% A/5.0% B within 1.1 min and maintained for 2.9 min. The flow velocity was 0.35 ml per minute. The column oven temperature was set to 40°C. The injection volume was 4 μl. The effluent was alternatively connected to an ESI–triple quadrupole linear ion trap (QTRAP)–MS.

Linear ion trap (LIT) and triple quadrupole (QQQ) scans were acquired using a triple quadrupole–linear ion trap mass spectrometer (QTRAP; AB4500 Q TRAP UPLC/MS/MS System) equipped with an electrospray ionization (ESI) turbo ion‐spray interface, which was operated in positive and negative ion modes and controlled by Analyst 1.6.3 software (AB Sciex). The ESI source operation parameters were as follows: ion source, turbo spray; source temperature, 550°C; ion‐spray voltage (IS), 5500 V (positive ion mode)/−4500 V (negative ion mode); ion source gas I (GSI), gas II (GSII), and curtain gas (CUR) were set to 50, 60, and 25.0 psi, respectively; collision‐activated dissociation (CAD) was high. Instrument tuning and mass calibration were performed using 10 and 100 μmol L^−1^ polypropylene glycol solutions in the QQQ and LIT modes, respectively. QQQ scans were acquired as multiple reaction monitoring (MRM) experiments with collision gas (nitrogen) set in the medium. Declustering potential and collision energy (DP and CE) for individual MRM transitions were calculated with further DP and CE optimization. A specific set of MRM transitions was monitored for each period according to the metabolites eluted within this period.

### 2.4 Qualitative and quantitative analyses of anthocyanin and flavonoid compounds

The ion current intensities and retention times were compared with those of our self‐developed “metware” database (MWDB database). Qualitative and quantitative analyses of the compounds were conducted according to the secondary spectrum information. The contents of different metabolites were analyzed according to the metabolite detection multimodal diagram.

### 2.5 Statistical analysis

Microsoft Excel 2010 and SPSS 21.0 software were used to process and analyze the data. Origin2018 was used to plot the data. Significant differences were accepted at *p* < .05 (*n* = 5). All data are expressed as the mean ± standard error.

## 3 RESULTS AND DISCUSSION

### 3.1 Effects of different iron treatments on the physicochemical properties of grape berries

Table 3 shows that the TSS and reducing sugar (RS) contents of grape berries increased under all of the iron treatments, where the highest TSS and RS contents were observed under FB3 (ferric citrate), which were 4.70% and 12.40% higher than those in the control, respectively. The TSS contents of grape berries did not differ significantly among the treatments, whereas the RS contents did. The RS contents were significantly higher under FB3 and FB5 (ferric sugar alcohol) than under the control and the other iron treatments, where the contents followed the order of: FB3 > FB5 > FB4 > FB2 > FB1. Except under FB3, the TAC values were lower under the iron treatments than under the control. The TAC value was the lowest under FB4 (ferric gluconate) (16.67% lower than that in the control). The iron treatments had significant effects on the sugar–acid ratio in grape berries. Compared with the control, the sugar–acid ratios were 14.63%, 8.12%, 4.47%, 20.28%, and 16.04% higher under FB1 (ferrous sulfate), FB2 (EDTA‐Fe), FB3, FB4, and FB5, respectively. The iron treatments also significantly increased the 100‐berry weight compared with the control, but the differences in the weights were not significant between the different iron treatments.

**TABLE 3:** Effects of different iron treatments on the physicochemical properties of grape berries

Index	Control	FB1	FB2	FB3	FB4	FB5
TSS	26.87 ± 0.05e	27.87 ± 0.06ab	27.67 ± 0.05bc	28.07 ± 0.10a	26.93 ± 0.09d	27.47 ± 0.02c
RS	17.84 ± 0.08c	17.86 ± 0.06c	18.70 ± 0.07b	20.06 ± 0.08a	18.91 ± 0.14b	19.82 ± 0.05a
TAC	0.84 ± 0.01a	0.76 ± 0.01bc	0.80 ± 0.01ab	0.84 ± 0.01a	0.70 ± 0.02d	0.74 ± 0.01cd
TSS/TAC	31.97 ± 0.21d	36.64 ± 0.12b	34.56 ± 0.14c	33.39 ± 0.11cd	38.45 ± 0.09a	37.09 ± 0.13ab
WB	94.78 ± 0.36c	115.42 ± 1.04a	110.66 ± 0.92b	117.05 ± 1.16a	113.63 ± 1.88ab	111.48 ± 0.98b

*Note*: Different lowercase letters indicate significant differences between treatments, according to Tukey's HSD (honest significant difference) test (*p* < .05).

Abbreviations: RS, reducing sugar (expressed as gram equivalent glucose L^−1^); TAC, titratable acid content (expressed as gram equivalent tartaric acid L^−1^); TSS, total soluble solids content (%); WB, weight of 100 berries (g).

### 3.2 Effects of different iron treatments on anthocyanin contents of grape peel

The UPLC–MS (ultrahigh performance–mass spectromtery) analysis detected 22 compounds (Table 4) in the grape peel, which mainly comprised cyanidins (five), paeoniflorins (four), petunidins (four), malvidins (five), delphinidins (three), and pelargonidin (one). The abbreviations used for these compounds are presented in Table 4. The contents of the anthocyanin monomers such as Cyacte, Dpacet, Cycoum, Mv, and Decoum were the highest in the peel. The iron treatments significantly increased the content of Mv and its various morphological derivatives, but did not have significant effects on the contents of the anthocyanins such as Pn, Pndigl, and Dp. The contents of Cy, Pt, Dp, and their derivatives in grape peel were significantly lower under the different iron treatments than in the control, except for FB3 and FB4. The contents of most anthocyanins were significantly higher under FB3 than under the other treatments, except for Decoum. The different iron treatments had significant effects on the total anthocyanin content of berry peel, where the anthocyanin content was the highest under FB3, which was 14.20%, 32.37%, 29.06%, 16.20%, and 35.16% higher than that in the control, FB1, FB2, FB4, and FB5, respectively.

**TABLE 4:** Effects of different iron treatments on contents of anthocyanins in grape peel

Flavonoids	Control	FB1	FB2	FB3	FB4	FB5
Cy	0.18 ± 0.02ab	0.08 ± 0.02c	0.12 ± 0.01bc	0.22 ± 0.02a	0.13 ± 0.02bc	0.09 ± 0.02c
Pl	4.15 ± 0.21b	2.93 ± 0.23c	3.19 ± 0.21bc	5.29 ± 0.48a	3.81 ± 0.61bc	2.80 ± 0.15c
Pn	3.28 ± 0.21b	3.02 ± 0.18b	3.35 ± 0.20b	4.80 ± 0.14a	3.67 ± 0.43b	3.03 ± 0.15b
Cygala	2.01 ± 0.06b	1.15 ± 0.06c	1.15 ± 0.08c	3.03 ± 0.20a	1.76 ± 0.33b	1.10 ± 0.05c
Cygluc	1.66 ± 0.03b	0.99 ± 0.06cd	0.89 ± 0.05d	2.65 ± 0.22a	1.41 ± 0.24bc	0.85 ± 0.08d
Pngluc	5.18 ± 0.12b	3.91 ± 0.22c	4.17 ± 0.33bc	6.74 ± 0.29a	4.49 ± 0.67bc	3.80 ± 0.21c
Mv	25.33 ± 0.47d	34.13 ± 0.55ab	36.50 ± 1.48a	32.00 ± 1.06bc	30.07 ± 0.66c	31.57 ± 1.30bc
Pt	8.37 ± 0.10ab	7.51 ± 0.27bc	7.16 ± 0.12c	8.66 ± 0.38a	7.58 ± 0.26bc	6.15 ± 0.40d
Cyacet	93.13 ± 3.37a	53.77 ± 2.49b	59.60 ± 5.37b	103.7 ± 4.23a	87.20 ± 13.06a	53.20 ± 4.33b
Pncaff	7.93 ± 0.50a	6.07 ± 0.10c	6.78 ± 0.49abc	8.11 ± 0.42a	7.69 ± 0.26ab	6.29 ± 0.81bc
Dpacet	47.70 ± 1.87a	37.63 ± 3.21b	35.07 ± 1.43b	51.10 ± 2.21a	48.20 ± 1.71a	37.27 ± 1.79b
Ptacet	12.93 ± 0.27a	10.83 ± 0.24bc	11.10 ± 0.47abc	12.77 ± 0.66ab	12.33 ± 0.83ab	9.27 ± 0.77c
Mvacet	11.77 ± 0.74b	13.6 ± 0.38a	13.80 ± 0.42a	12.40 ± 0.35ab	13.03 ± 0.15ab	13.43 ± 0.72ab
Mvmalo	1.80 ± 0.13c	2.42 ± 0.06ab	2.76 ± 0.15a	1.96 ± 0.16bc	2.38 ± 0.14ab	2.04 ± 0.17bc
Cycoum	30.83 ± 1.45b	20.3 ± 1.37c	20.37 ± 1.13c	56.83 ± 2.89a	27.03 ± 3.09b	19.10 ± 1.31c
Decoum	23.5 ± 1.29a	15.40 ± 1.06b	14.4 ± 0.15b	21.27 ± 1.57a	15.83 ± 0.47b	16.37 ± 0.23b
Ptarab	0.15 ± 0.02c	0.18 ± 0.01bc	0.19 ± 0.01abc	0.19 ± 0.01abc	0.24 ± 0.03a	0.22 ± 0.01ab
Ptcoum	20.70 ± 0.75b	17.23 ± 0.43c	17.60 ± 0.20c	25.07 ± 1.12a	21.50 ± 1.51b	16.90 ± 0.25c
Pndigl	2.87 ± 0.11a	2.05 ± 0.03a	2.18 ± 0.23a	2.71 ± 0.20a	2.82 ± 0.61a	2.26 ± 0.21a
Dp	18.03 ± 0.29ab	17.77 ± 0.54ab	22.10 ± 2.52a	16.07 ± 0.17b	20.8 ± 0.93a	15.00 ± 1.80b
cMvcoum	3.35 ± 0.09b	3.35 ± 0.03b	3.14 ± 0.11b	3.16 ± 0.14b	4.05 ± 0.25a	3.33 ± 0.10b
Mvdi	1.77 ± 0.18c	2.97 ± 0.23b	4.2 ± 0.17a	1.88 ± 0.27c	2.99 ± 0.18b	2.61 ± 0.28b
Total anthocyanins	326.45 ± 4.13b	257.33 ± 5.92c	269.92 ± 7.25c	380.50 ± 10.69a	318.87 ± 19.88b	246.71 ± 11.65c

*Note*: Different lowercase letters indicate significant differences between treatments, according to Tukey's HSD (honest significant difference) test (*p* < .05).

Abbreviations: Cy, cyanidin; Pl, pelargonidin‐3‐O‐glucoside; Pn, peonidin‐3‐O‐arabinoside; Cygala, cyanidin‐3‐O‐galactoside*; Cygluc, cyanidin‐3‐O‐glucoside (kuromanin)*; Pngluc, peonidin‐3‐O‐glucoside; Mv, malvidin‐3‐O‐arabinoside; Pt, petunidin‐3‐O‐glucoside; Cyacet, cyanidin‐3‐O‐(6′′‐O‐acetyl)glucoside; Pncaff, peonidin‐3‐O‐(6′′‐O‐acetyl)glucoside; Dpacet, delphinidin‐3‐O‐(6′′‐O‐acetyl)glucoside; Ptacet, petunidin‐3‐O‐(6′′‐O‐acetyl)glucoside; Mvacet, malvidin‐3‐O‐(6′′‐O‐acetyl)glucoside; Mvmalo, malvidin‐3‐O‐(6′′‐O‐malonyl)glucoside; Cycoum, cyanidin‐3‐O‐(6′′‐O‐p‐coumaroyl)glucoside; Decoum, delphinidin‐3‐O‐(6′′‐O‐p‐coumaroyl)glucoside; Ptarab, petunidin‐3‐O‐glucoside‐5‐O‐arabinoside; Ptcoum, petunidin‐3‐O‐(6′′‐O‐p‐Coumaroyl)glucoside; Pndigl, peonidin‐3,5‐O‐diglucoside; Dp, delphinidin‐3,5‐di‐O‐glucoside; cMvcoum, malvidin‐3‐O‐(6′′‐O‐p‐coumaroyl)glucoside; Mvdi, malvidin‐3,5‐di‐O‐glucoside (malvin).

### 3.3 Effects of different iron treatments on flavanols and flavonols in grape peel

The flavonoids detected in grape berry skin are shown in Table 5, where 19 flavanols and 42 flavonols were detected. The abbreviations for these compounds are listed in Table 5 (only some representative data are presented in the table). Flavanols included catechins, epicatechins, and gallocatechins, while flavanones included quercetin, myricetin, kaempferol, and 6‐hydroxy kaempferol derivatives. In particular, Ca, Hydro, Ga, Cid, and αCadih were the main flavanols detected. The content of most individual flavanols was higher in grape skin under FB3 than in the control, except for Hydro and Ga. However, the contents of Hydro and Ga were significantly higher under FB1, FB4, and FB5 compared with the control, where the Hydro content was the highest under FB1, i.e., 4.08% and 15.91% higher than those under the control and FB3, respectively. The Ga content was the highest under FB5, as it was 28.39% and 42.82% higher than those under the control and FB3, respectively. The total individual flavanol contents of the grape peel were lower under the iron treatments than under the control, except for FB3. The total individual flavanol content was the lowest under FB2, where it was 23.12% lower than that under the control. However, the total individual flavanol contents did not differ significantly under FB1, FB4, and FB5, where they were 9.31%, 4.80%, and 9.31% lower, respectively, than that under the control.

**TABLE 5:** Effects of different iron treatments on the flavanol and flavonol contents of grape peel

Flavonoid	Control	FB1	FB2	FB3	FB4	FB5
Ca	3.73 ± 0.11b	3.11 ± 0.16c	2.58 ± 0.13d	4.84 ± 0.10a	2.98 ± 0.07cd	3.36 ± 0.19bc
Hydro	2.45 ± 0.08ab	2.55 ± 0.21a	2.53 ± 0.06a	2.20 ± 0.03c	2.36 ± 0.08b	2.33 ± 0.10b
Ga	4.65 ± 0.15cd	5.42 ± 0.10b	4.73 ± 0.13c	4.18 ± 0.02d	5.09 ± 0.32bc	5.97 ± 0.07a
Cid	7.31 ± 0.62b	5.87 ± 0.50bc	4.30 ± 0.20d	9.46 ± 0.47a	7.00 ± 0.69b	5.14 ± 0.23cd
αCadih	5.87 ± 0.21b	4.28 ± 0.03c	3.24 ± 0.06d	7.16 ± 0.41a	5.05 ± 0.49bc	4.20 ± 0.13c
Total Flavan‐3‐ols	33.30 ± 1.04b	30.20 ± 0.68b	25.60 ± 0.42c	38.76 ± 1.04a	31.70 ± 1.65b	30.20 ± 0.82b
Ka	18.77 ± 0.87a	17.5 ± 1.56a	17.23 ± 0.74a	16.43 ± 1.69ab	12.05 ± 2.53b	17.13 ± 0.03a
Qugluco	16.3 ± 0.00ab	16.53 ± 1.21a	17.23 ± 0.67a	17.13 ± 0.87a	13.60 ± 1.11b	15.57 ± 0.56ab
sQuglu	20.63 ± 0.45a	19.33 ± 0.58ab	20.97 ± 1.14a	20.00 ± 0.90ab	16.43 ± 2.02b	18.40 ± 1.31ab
My	17.5 ± 0.53b	19.77 ± 0.79ab	21.97 ± 1.42a	19.73 ± 1.52ab	17.73 ± 0.65b	17.30 ± 0.66b
rKaglu	42.3 ± 0.60b	25.63 ± 0.72d	26.10 ± 1.72d	72.93 ± 3.29a	32.30 ± 2.86c	23.97 ± 1.09d
Kaneo	40.37 ± 2.08b	25.27 ± 1.17c	25.27 ± 2.29c	68.9 ± 6.29a	34.53 ± 4.63bc	24.9 ± 0.70c
*rQuglu	25.57 ± 1.25a	16.53 ± 1.05c	16.20 ± 0.55c	22.43 ± 0.98b	16.47 ± 0.24c	18.60 ± 0.55c
Qu	24.57 ± 1.33a	16.00 ± 0.79c	16.33 ± 0.37c	21.43 ± 0.98b	16.50 ± 0.38c	17.57 ± 0.24c
Quneo	21.90 ± 0.83a	14.70 ± 1.10b	15.53 ± 0.22b	21.37 ± 0.59a	15.33 ± 0.50b	16.63 ± 0.77b
rQuglu	25.57 ± 0.76a	16.67 ± 1.09c	16.67 ± 0.79c	21.67 ± 1.22b	16.50 ± 0.56c	17.80 ± 0.55c
Se	19.53 ± 0.83b	16.3 ± 0.67c	15.30 ± 0.93c	24.50 ± 1.18a	21.83 ± 1.12ab	16.30 ± 0.79c
Me	15.40 ± 1.21bc	13.37 ± 0.52c	13.43 ± 0.12c	23.57 ± 1.04a	18.03 ± 1.26b	13.17 ± 0.71c
Total flavonoids	439.41 ± 8.19b	349.16 ± 13.68c	370.82 ± 8.02c	491.88 ± 18.92a	345.96 ± 17.98c	351.03 ± 5.32c

*Note*: Different lowercase letters indicate significant differences between treatments, according to Tukey's HSD (honest significant difference) test (*p* < .05).

Ca, catechin*; Hydro, 4′‐hydroxy‐5,7‐dimethoxyflavanone; Ga, gallocatechin*; Cid, cinchonain Id; αCadih, catechin‐(7,8‐bc)‐4α‐(3,4‐dihydroxyphenyl)‐dihydro‐2‐(3H)‐one. Ka, kaempferol‐7‐O‐glucoside*; Qugluco, quercetin‐7‐O‐glucoside*; sQuglu, quercetin‐4′‐O‐glucoside (Spiraeoside)*; My, myricetin‐3‐O‐glucuronide; rKaglu, kaempferol‐3‐O‐glucoside‐7‐O‐rhamnoside; Kaneo, kaempferol‐3‐O‐neohesperidoside; *rQuglu, quercetin‐3‐O‐glucoside‐7‐O‐rhamnoside; Qu, quercetin‐7‐O‐rutinoside; Quneo, quercetin‐3‐O‐neohesperidoside; rQuglu, quercetin‐3‐O‐(4′′‐O‐glucosyl)rhamnoside; Se, sexangularetin‐3‐O‐glucoside‐7‐O‐rhamnoside; Me, 6‐C‐methylquercetin‐3‐O‐rutinoside.

Different iron treatments affected flavonol content. The contents of 12 monomer flavonols, including Ka, Qugluco, sQuglu, My, rKaglu, Kaneo, * rQuglu, Qu, Quneo, rQuglu, Se, and Me, were the highest in grape peel. The contents of the two monomeric flavonol compounds, rKaglu and Kaneo, were the highest, and their levels also differed significantly among the iron treatments. The rKaglu and Kaneo contents were the highest under FB3, where they were 72.41% and 70.67% higher than those of the control, respectively. The rKaglu and Kaneo contents were the lowest under FB5 (43.33% and 38.32% lower, respectively), compared with the control. The contents of other flavonol monomers also varied greatly among different treatments, where the levels of zero, 10, 14, two, zero, and 16 individual flavonols were the highest in FB1, FB2, FB3, FB4, FB5, and the control, respectively.

### 3.4 Principal component analysis of grape berry flavonoids

**FIGURE 1:**
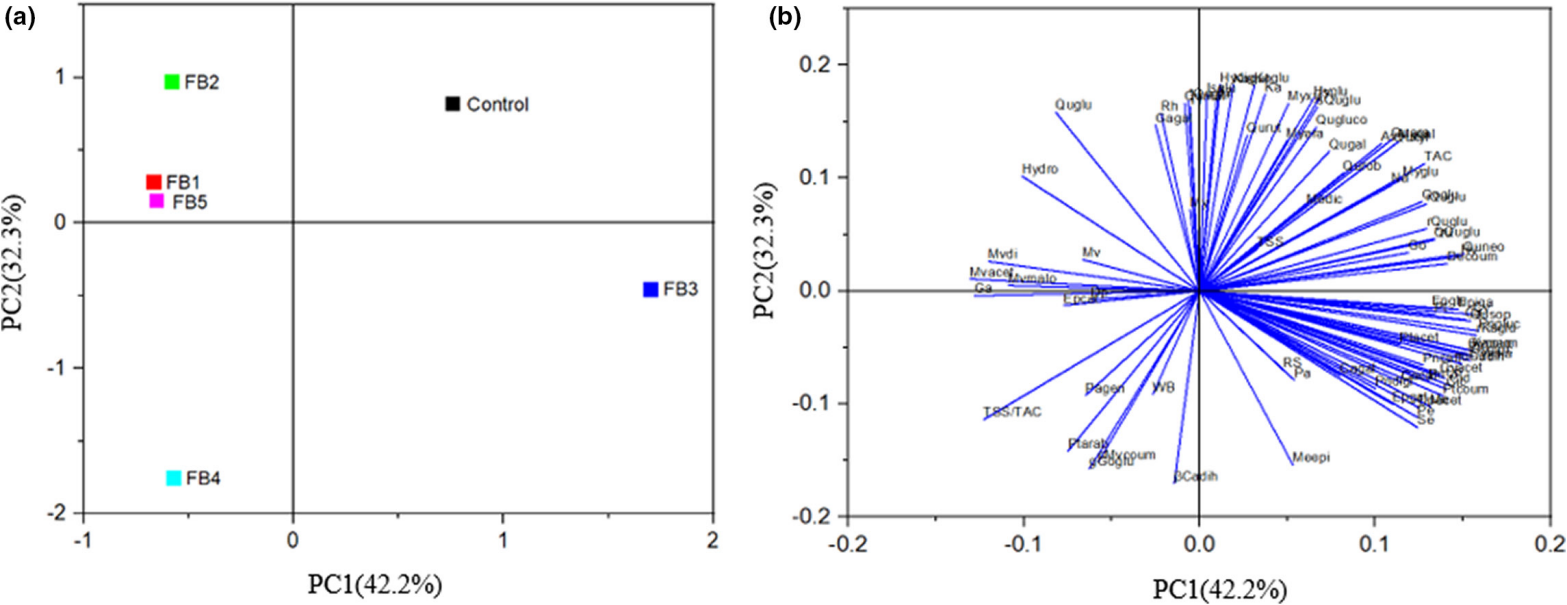
Principal component analysis (PCA) results obtained based on the correlation matrix for the physical and For Review Only chemical indexes and flavonoid components of grapes: (a) scoring plot and (b) loading plot. The abbreviations used in (b) are defined in Tables 3, 4, and 5

The different iron treatments had significant effects on the content and proportion of sugar acids and flavonoids in grape berries. Principal component analysis (PCA) was conducted to determine the overall differences in flavonoids under different iron treatments. Figure 1 shows that the first two PCs accounted for 42.20% and 32.30% of the variance, respectively, and thus, 74.50% of the total variance. PC1 was mainly explained by flavanones such as Qu, Se, and Pt, and PC2 was mainly explained by anthocyanins and flavanols such as My and Qu. The scoring plot in Figure 1a shows that the results obtained under the different iron treatments and the control were clearly separated, with FB1, FB2, and FB5 in a single cluster, and the control, FB3, and FB4 in three separate clusters. The loading plot in Figure 1b shows that the sugar content and the contents of Mv, Pt, Ca, Ep, Qu, and their derivatives were well separated in the control and iron treatment groups, thereby indicating that the iron treatments affected the sugar, acid, and flavonoid contents of grape berries. However, FB1, FB2, and FB5 were grouped in the same cluster (A1), thereby indicating that the effects of these three iron treatments on the quality indices for grape berries and flavonoid contents were not significantly different, and that these treatments increased the contents of Qugal, Ga, Mymal, Mv, and their derivatives. FB4 clustered separately (A2) and TSS/TAC, WB, and the content of flavanols, such as gGoglu, increased under ferric gluconate (FB4) treatment. FB3 was within the confidence interval (A3), showing that RS and almost all flavonoids in grape berries were increased under ferric citrate treatment.

## 4 DISCUSSION

Alvarez‐Fernàndez et al., (2006) showed that iron deficiency decreases berry sugar content. The sugar–acid ratio is generally a measure of berry ripeness. In the present study, spraying with different forms of iron improved the quality of wine grapes, where TSS increased, and sugar content and TAC decreased, thereby increasing the sugar–acid ratio. The increase in the sugar–acid ratio in grape berries under the iron treatments indicates that iron can promote berry ripening, as also observed in pears (“Deveci” and “Santa Maria”) (Ozturk et al., 2019), table grapes (cv. “Thompson Seedless”) (Taghavi et al., 2020), and wine grapes (*Vitis vinifera* cv.) (Shi et al., 2017). Reductions in RuBisCO (ribulose‐1,5‐bisphosphate carboxylase‐oxygenase) activity levels and lower chlorophyll and carotenoid contents in iron‐deficient plants lead to a lower leaf CO_2_ exchange rate and photosynthetic efficiency, which may explain the higher sugar content under iron treatment in the present study (Chen et al., 2004). We found that the glycolic acid contents of grape berries differed significantly among the iron treatments, while the TSS contents were also higher and the sugar contents were lower under FB2, FB3, FB4, and FB5 than under FB1. Organic chelated iron is a small molecule that is absorbed by the leaves when chelated by sugar alcohols and amino acids, thereby avoiding the oxidation and precipitation of ferrous sulfate when sprayed alone (FB1), facilitating the absorption of nutrient iron (Fernández & Ebert, 2005). In addition, sugar alcohols and amino acids are small organic molecules with good moisture retention, permeability, and ductility characteristics; thus, they can reduce the surface tension and improve the capacity of leaf surfaces to absorb iron (Singh et al., 2013). Tartaric acid and malic acid are the main organic acids present in grape berries. We found that iron treatment decreased TAC, except for FB3 (ferric citrate), possibly because external application of iron promoted the accumulation of sugars and accelerated berry ripening, whereas the malic acid content of berries gradually decreased as the berry matured (Karimi et al., 2019). Malic and citric acids are the main substrates for plant respiration. The TSS and reducing sugar contents of berries increased under FB3 (ferric citrate), whereas TAC did not change compared with the control, probably because the externally applied ferric citrate was consumed by respiration to decrease the decomposition of malic acid; thus, TAC was higher under FB3 (ferric citrate) compared with other iron treatments (Chen et al., 2004; Schlegel et al., 2006). The weight of berries is determined by their size and density, which are important factors that affect the quality of grapes. The weights of the grape berries were significantly higher under the iron treatments than under the control, possibly because iron increased the metabolic activity of the plants. Iron deficiency during grape growth is known to reduce membrane integrity, leaf CO_2_ content, exchange rate, and chlorophyll photosynthetic efficiency to inhibit the accumulation of dry matter, which may explain why iron treatment significantly increased the weight of the berries in the present study (Bertamini & Nedunchezhian, 2005).

The types and quantities of anthocyanins detected in grape peel in the present study were generally consistent with those previously reported (Arozarena et al., 2002; Mattivi et al., 2006; Shi et al., 2017). Anthocyanins are important pigments in red grape. The proportions and quantities of anthocyanins were determined based on the specific variety and cultivation conditions. Studies have shown that iron is an important factor affecting anthocyanin synthesis (Ahmed et al., 1997). In the present study, we found that spraying different forms of iron increased the content of specific anthocyanins. In particular, foliar application of ferric citrate (FB3) and ferric gluconate (FB4) significantly increased the contents of some individual anthocyanins and the total anthocyanins in the grape peel, probably because glucose, fructose, and sucrose can induce the accumulation of anthocyanin in grape berries (Zheng et al., 2009). Indeed, the sugar content of grape berries increased significantly under these two treatments, thereby promoting anthocyanin synthesis in grape peel. However, we also found that under treatment with ferrous sulfate (FB1), EDTA‐Fe (FB2), and ferric sugar alcohol (FB5), the levels of some individual anthocyanins (Cyacet, Dpacet, and Cycoum) were lower than those in the control, resulting in the total anthocyanin content being lower than that in the control as well. In contrast, previous studies found that anthocyanin content increased under iron treatment (Ahmed et al., 1997; Singh et al., 2013), where different iron treatments significantly increased the content of Mv and its morphological derivatives. Cy and Dp are considered the precursors of Pn, Mv, and Pt (He et al., 2010), respectively. Iron treatment led to an increase in the contents of Mv and its morphological derivatives, which may explain the decrease in the contents of Cy and Dp. Anthocyanins that contain more methoxy groups in the B ring may contribute to the redness of grapes. Methylated anthocyanins, including Pn, Mv, and their derivatives, are relatively stable and are the main anthocyanins in mature grape berries (He et al., 2012). Thus, anthocyanins, such as Pn and Mv, have very important effects on wine grapes (De Gaulejac et al., 2001). Therefore, iron is important for improving grape berry quality.

Flavanols and flavonols are subclasses of flavonoids. The types and quantities of flavanols and flavonols detected in grape peel in the present study were generally consistent with those reported by Mattivi et al. (2006). Grape peel is the main site of flavonoid synthesis and the main source of flavonoids in wine (Gonzalez‐Manzano et al., 2009). In this study, we found that the Hydro and Ga contents of grape skins were higher when the leaves were sprayed with ferrous sulfate (FB1), EDTA‐Fe (FB2), ferric gluconate (FB4), and ferric glycol (FB5) compared with the control, and the total flavanol content was higher when treated with ferric citrate (FB3). Thus, the foliar application of iron affected the flavanol content of grape skins. However, there were differences in the results of the different treatments, which may be due to two reasons. First, Fe^2+^ is the most important factor affecting flavanol synthesis (Perron & Brumaghim, 2009; Zeng et al., 2019). Ferrous sulfate is easily oxidized to ineffective iron in the air, and the organic compound iron is not as stable as the chelated iron. In the process of iron ions being absorbed by plants through osmosis, chelated iron will not dissolve on the surface of the cuticle, and the chelate will reduce the permeability of calcium ions in the cuticle by a factor of 7; thus, iron is more easily absorbed by plants. Second, the absorption and utilization of iron by plants depend on substances such as siderophores and alkyl glucosides in surfactants. Ferric citrate surfactants and other compounds can significantly improve the efficiency of foliar fertilizers. Flavonoids are present in most higher plants and are products of flavonoid biosynthesis, while flavonols are closely related to anthocyanin biosynthesis (Gonzalez‐Manzano et al., 2009; Jaakola, 2013; Mattivi et al., 2006). We found that the different iron treatments had significantly different effects on the contents of specific flavonols in a manner similar to the changes in the anthocyanin content, possibly because the enzymes involved in the production of flavonols overlap greatly with those involved in the production of anthocyanins (Gonzalez‐Manzano et al., 2009). In addition, these classes of compounds share the same skeleton and differ only in the oxidation state of the central pyran ring (Jaakola, 2013). Alvarez‐Fernandez et al. (2011) found that iron treatment can improve the photosynthetic efficiency of grape vines and affect the synthesis of phenolic compounds or other secondary metabolites using precursors. However, further research is required to understand why different forms of iron can have different effects on the phenolic compound content in grape peel.

## 5 CONCLUSION

In this study, we found that five different foliar iron treatments affected the fructose, acid, and flavonoid contents of Cabernet Sauvignon grapes, and that the various iron treatments also had different effects. Spraying iron on the leaves could increase the sugar content and reduce the acid content of berries. However, spraying ferrous sulfate, EDTA‐Fe, ferric gluconate, and ferric sugar alcohol on the leaves reduced the total anthocyanin, flavanol, and flavonol contents in the peel. In addition, the contents of specific flavonoid monomers were significantly higher in the grape peel under some iron treatments than in the control, as well as with the other iron treatments. However, our comprehensive study showed that foliar spraying with ferric citrate balanced the sugar–acid ratio in the berry and increased the anthocyanin, flavanol, and flavonol contents of the grape peel to further improve the quality of the grapes, thereby possibly enhancing the overall nutritional content of the berries and the final wine quality.

## Data Availability

The data that support the findings of this study are available from the corresponding author upon reasonable request.
